# A Case of Lelliottia amnigena-Induced Acute Calculous Cholecystitis and a Literature Review

**DOI:** 10.7759/cureus.82743

**Published:** 2025-04-21

**Authors:** Dimitrios Raptis, Amr Aljareh, Usman Shah, Muhammad Umer Tufail, Shiny Teja Kolli

**Affiliations:** 1 Internal Medicine, New York City Health and Hospitals/Jacobi/North Central Bronx, The Bronx, USA

**Keywords:** acute calculous cholecystitis, hepatitis a virus (hav), lelliottia amnigena, opportunistic bacterial infection, refractory septic shock

## Abstract

Atypical presentations can confuse the clinical picture, especially in patients with comorbidities. Particularly, rare pathogens, such as *Lelliottia amnigena,* can cause infections in immunocompromised patients or, sometimes, present as epidemic infections. We present a case of a female patient with a history of cryptogenic cirrhosis and poorly controlled diabetes, who developed acute calculous cholecystitis with positive bile cultures for *L. amnigena*, a rare, Gram-negative facultative anaerobic bacillus, usually associated with water sources and immunocompromised hosts. The patient also had positive titers for acute hepatitis A. This case is the first to our knowledge that reports *L. amnigena*-induced acute cholecystitis, along with concomitant acute hepatitis A positive serology. We aim to emphasize the importance of a broad differential diagnosis in such patients, early identification of rare pathogens, and the role of comprehensive clinical judgment in guiding treatment decisions.

## Introduction

In real-time clinical practice, the combination of multiple comorbidities, atypical presentations, and rare infections often leads to diagnostic conundrums that challenge even the most experienced clinicians. Infectious agents, and especially multidrug-resistant ones, have emerged over the last decades, partially due to extensive antibiotic use. Clinical judgement is always warranted in case of rare opportunistic infections with pathogens that usually affect immunocompromised hosts.

*Lelliottia amnigena, *a rare Gram-negative facultative anaerobic bacillus, has been described in the past as a cause of various infections, including bloodstream infections, endophthalmitis, or wound infections, sometimes in immunocompromised individuals. To our knowledge, there is no report of acute purulent calculous cholecystitis in the setting of this bacterium.

We present here a case of acute purulent calculous cholecystitis with bile cultures growing *L. amnigena*, along with concomitant positive titers for acute hepatitis A. The unexpected co-occurrence of *L. amnigena* and positive serology for acute hepatitis A indicates the complexity of infectious diseases and the unpredictable nature of severe illnesses in immunocompromised individuals. Through an extensive literature review, we also offer a comprehensive overview of previously described infections caused by *L. amnigena*.

This case was previously presented as a poster during the Annual Scientific Meeting and Postgraduate Course of the American College of Gastroenterology in Philadelphia, PA, on October 28, 2024.

## Case presentation

An 80-year-old female, who carried a history significant for cryptogenic cirrhosis with evidence of portal hypertension, as well as poorly controlled type 2 diabetes mellitus on metformin, with latest glycated hemoglobin of 9.5%, presented with right facial droop, with suspicion of acute stroke. Despite the neurological symptoms, she had no apparent signs of distress. The rest of the physical examination did not reveal any remarkable findings; her abdomen was soft and non-tender. Initial chest imaging suggested a possible lower respiratory infection, necessitating empiric treatment for community-acquired pneumonia with azithromycin and ceftriaxone on the second day of admission, since the patient had subtle symptoms of lower respiratory, including a mild cough. Her facial droop had, meanwhile, resolved, and because of the negative head imaging, it was attributed to a transient ischemic attack (TIA). However, the patient started exhibiting signs of septic shock, characterized by increased lactate, severe acidosis, and hemodynamic instability (Table 1).

**Table 1:** Laboratory findings on presentation and two days after. Abbreviations: ALK PHOS, alkaline phosphatase; AST, aspartate aminotransferase; ALT, alanine aminotransferase; BUN, blood urea nitrogen; HbA1c, glycated hemoglobin; WBC, white blood cell count

Parameter	Baseline	2 Days After	Reference Range
HbA1c	9.5	—	4.0–5.6%
Lactate	8.9	13.6	0.3–1.3 mmol/L
WBC	16.30	23.53	3.5–11.0 × 10⁹/L
Procalcitonin	0.45	0.31	0.02–0.08 ng/mL
AST	40	>7,000	1–40 U/L
ALT	17	3,436	1–40 U/L
ALK PHOS	171	284	35–104 U/L
BUN	44	51	5–26 mg/dL
Creatinine	2.3	2.6	0.5–1.5 mg/dL

**Figure 1:**
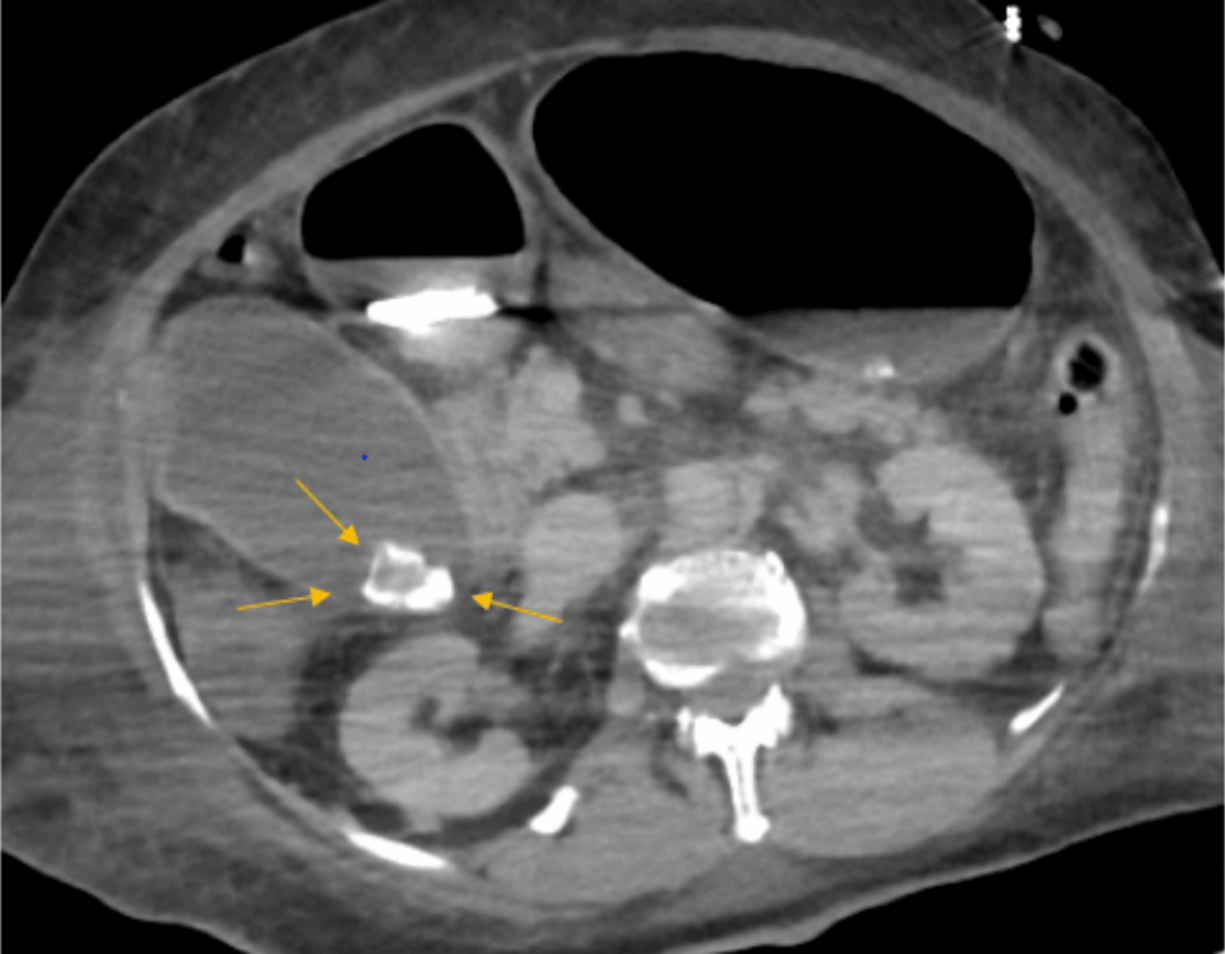
Computed tomography (CT) scan of the abdomen. Distended gallbladder with multiple gallstones with wall thickening, indicative of acute calculus cholecystitis.

Concurrently, acute oliguric kidney injury required the initiation of continuous renal replacement therapy (CRRT). Subsequent computed tomography (CT) of the abdomen, performed due to an unidentified source of septic shock, not clearly explained by the subtle findings of possible pneumonia on chest radiology, revealed acute calculous cholecystitis (Figure 1). The patient's condition became more critical than her initial presentation suggested, with the patient needing vasopressors. Blood and urine cultures repeatedly came back negative. The identification of *Enterobacter amnigenus *biogroup 2 (currently known as *L. amnigena*) in the cholecystitis fluid, drained through interventional radiology (IR) assistance, was surprisingly the most apparent source of her septic shock, prompting empiric broad-spectrum antibiotic coverage. Further testing due to marked liver function impairment, which was initially attributed to shock liver, revealed positive IgM for hepatitis A virus (HAV), further complicating her diagnostic approach.

The patient was maintained on piperacillin-tazobactam and vancomycin after her deterioration, but before she was on ceftriaxone and azithromycin for suspected community-acquired pneumonia. Although the identified bacterium was sensitive to ceftriaxone, as per the laboratory, it is reported to be known to possess inducible beta-lactamases, which cause failure with third-generation cephalosporin or beta-lactamase therapy despite in vitro susceptibility. The antibiogram that followed the culture indicated resistance to cefazolin, cefoxitine, intermediate resistance to levofloxacin, and at least in vitro sensitivity to piperacillin/tazobactam, ampicillin/sulbactam, ceftazidime, and ceftriaxone.

To our knowledge, this is the first case report involving *L. amnigena* in acute calculous purulent cholecystitis. We want to emphasize the various diagnostic challenges that may arise from managing critically ill patients with equivocal presentations. This patient's clinical course shows the importance of an accurate clinical judgement, along with a comprehensive approach to every patient, even with obvious presentations. It also enforces the need for awareness by clinicians of rare bacterial factors, such as *L. amnigena*, as possible source of severe infections.

## Discussion

Initially named *E. amnigenus* in 1981 [1], this Gram-negative, facultative anaerobic bacillus is now known as *L. amnigena*, a nomenclature proposed by Brady et al. in 2013 [2]. The term “amnigena” means “born in a river” and is probably pointing toward the origin of this bacterium from the water. It was identified from water sources and later from food [3] and is a potential pathogen in humans, especially immunocompromised individuals. There have been a few reports from around the world, underlining its potential pathogenicity, with paradigms ranging from endophthalmitis and bloodstream infections to wound, respiratory tract, and urinary tract infections.

Back in 1991, a case report described the isolation of *E. amnigenus* from a central venous line catheter tip and later from a blood culture in a heart transplant recipient, suggesting its involvement in bloodstream and catheter-associated infections, particularly in patients undergoing immunosuppressive therapy, since that patient was on treatment with anti-rejection medications [4]. Additionally, a recent case report in 2023 described the respiratory coinfection of *L. amnigena*, along with *Pseudomonas putida* in a critically ill patient with SARS-CoV-2 infection, thus showing its potential to complicate other disease courses, mainly in individuals with underlying health conditions, since this patient had history of diabetes, chronic obstructive pulmonary disease, bronchiectasis, otitis externa with *Candida parapsilosis* and pulmonary actinomycosis [5].

Accordingly, *L. amnigena* has been described as a potential cause of “epidemic” infections, as reported in a case series from India, where 19 out of 63 patients who underwent cataract surgery in a center developed endophthalmitis - a severe intraocular infection - requiring antibiotic management. The disease spread was attributed to poor quality of aseptic precautions during the surgery [6]. The same type of infection, though, has also been described in an immunocompetent young individual [7]. These findings show that this bacterium can also affect healthy individuals without other comorbidities.

*L. amnigena *has also been found as the infectious agent in an individual who suffered a motor vehicle injury with significant injuries to his feet. In particular, two weeks after receiving intravenous antibiotics, he suffered a below-the-knee amputation because of the severity of the infection, with cultures from local wounds growing *L. amnigena*, *Leclercia adecarboxylata*, and eventually *Absidia *spp [8].

Interestingly, although with a low incidence of detection, *L. amnigena* was found as a pathogen in just one out of 137 positive blood cultures from septic patients, in a multicenter study in Ethiopia [9]. According to this, it was hardly identified as a co-infectious agent in swabs taken from lesions from patients with tungiasis, a parasitic skin disease, in a study in Western Kenya, since it was among the less frequent pathogens causing secondary infection in those patients [10] (Table 2).

**Table 2:** Examples of infections by Lelliottia amnigena that have been reported.

Infection Type	Description	Patient population
Endophthalmitis [6,7]	Severe intraocular infection, sometimes following surgical procedures like cataract surgery	Patients undergoing eye surgery
Bloodstream or catheter-associated Infections [4,9]	Infections are particularly in immunocompromised individuals, such as transplant recipients	Organ transplant recipients, patients on immunosuppressive therapy
Wound Infections [8,10]	Infections following traumatic injury	Trauma patients, such as those from motor vehicle accidents
Respiratory Tract Infections [5]	Complicating existing respiratory conditions, especially in individuals with lung diseases	Patients with chronic lung diseases

In this case, we encountered a patient with an unusual presentation, initially managed for TIA and later on, for septic shock with broad-spectrum antibiotics and CRRT for acute renal failure and severe lactic acidosis. Cultures from the incidentally found calculous cholecystitis grew *L. amnigena*, which required aggressive therapy with broad-spectrum antibiotics and vasopressors. The patient was, at the same time, found to have possible acute hepatitis A infection, since titers for acute infection were positive on two consecutive lab tests. It is already described that HAV can cause acalculous cholecystitis [11-13], but there are no reported cases of calculous cholecystitis to our knowledge, so it is unclear whether hepatitis A triggered the cholecystitis in this case. Furthermore, liver cirrhosis is considered generally a risk factor for cholelithiasis, although some of the etiologies of cirrhosis might be more strongly related to gallbladder disease, such as hepatitis C-induced cirrhosis [14,15]. In our case, it is not clear whether the incidental cholelithiasis was related to the cirrhosis history. However, it is very possible that cirrhosis itself was a significant risk factor that finally led to a severe opportunistic infection in this patient.

This is the first report, to our knowledge, that poses a possible relationship between positive serology for acute hepatitis A infection and calculous cholecystitis, along with a very rare opportunistic pathogen, which has not been described in the past to cause acute cholecystitis, to our knowledge. However, it is still possible that the suspected acute hepatitis A might have just been an incidental, irrelevant infection and not the real trigger for the acute purulent cholecystitis in this patient, which might have just been the *L. amnigena *infection. Further studies are warranted to further support *L. amnigena* and/or acute HAV infection as a causative agent of acute calculous cholecystitis.

## Conclusions

This case emphasizes how unpredictable can the nature of a disease in critically ill patients sometimes be, where a seemingly straightforward presentation can quickly progress into a complex, multifaceted clinical picture. What started as a suspicion of stroke rapidly evolved into septic shock, renal failure, and ultimately a rare *L. amnigena* infection complicating an acute calculus cholecystitis. Furthermore, the unexpected twist of acute hepatitis A serology likely influenced the course of illness. We present this case in an effort to raise suspicion that, even with common comorbidities, such as diabetes and cirrhosis, rare infections can emerge and significantly alter a patient’s course. The need for a broad, flexible diagnostic approach is essential to uncover underlying, and often rare, causes. In an age of increasingly resistant pathogens, *L. amnigena* exemplifies how lesser-known bacteria can play insidious roles in critical care settings. Moving forward, awareness of atypical manifestations of bacterial co-infections will be crucial in improving patient outcomes.
